# National Veterinary College

**Published:** 1896-10

**Authors:** 


### NATIONAL VETERINARY COLLEGE.

THIS college, as heretofore announced, now becomes the Veterinary Department of the Columbian University, with a curriculum of three years of six months each. The Rev. B. L. Whitman, D.D., President of the Columbian University, becomes the central head. William P. Carr, M.D., has been added as professor of general physiology. E. A. de Schweinitz, A.M., Ph.D., M.D., enters upon the duties of professor of chemistry in place of Edwin R. Hodge, M.D., resigned. D. E. Buckingham, V.M.D., succeeds as professor of materia medica and therapeutics, J. W. Hodges, M.D. James Carroll, M.D., succeeds Veranus A. Moore, B.Sc., M.D., as professor of pathology and bacteriology. Cecil French, D.V.S., has been added to the staff as professor of canine pathology. The chair of anatomy is unfilled owing to the retirement of William Fink, D.V.S. W. S. Washburn, M.D., will deliver the course on histology, succeeding Charles R. Clarke, M.D. Charles J. Hadfield, D.V.S., one of the special lecturers of the preceding year, has been added to the faculty and will fill the rôle of demonstrator of anatomy. B. E. Harper, D.V.S., R. H. Hadfield, D.V.S., and C. H. Lockwood, D.V.S., have also been added as assistant demonstrators. To the hospital staff as visiting and consulting surgeons Drs. D. E. Buckingham and Cecil French.

